# Utilization of antihypertensive drugs in obesity-related hypertension: a retrospective observational study in a cohort of patients from Southern Italy

**DOI:** 10.1186/s40360-016-0055-z

**Published:** 2016-03-16

**Authors:** Mauro Cataldi, Ornella di Geronimo, Rossella Trio, Antonella Scotti, Andrea Memoli, Domenico Capone, Bruna Guida

**Affiliations:** Division of Pharmacology, Department of Neuroscience, Reproductive and Odontostomatologic Sciences, Federico II University of Naples, Via Pansini n°5, Naples, 80131 Italy; Division of Physiology, Department of Clinical Medicine and Surgery, Federico II University of Naples, Naples, Italy; Division of Nephrology, Department of Public Health, Federico II University of Naples, Naples, Italy

## Abstract

**Background:**

Although the pathophysiological mechanisms of arterial hypertension are different in obese and lean patients, hypertension guidelines do not include specific recommendations for *obesity*-*related hypertension* and, therefore, there is a considerable uncertainty on which antihypertensive drugs should be used in this condition. Moreover, studies performed in general population suggested that some antihypertensive drugs may increase body weight, glycemia and LDL-cholesterol but it is unclear how this impact on drug choice in clinical practice in the treatment of obese hypertensive patients. Therefore, in order to identify current preferences of practitioners for obesity-related hypertension, in the present work we evaluated antihypertensive drug therapy in a cohort of 129 pharmacologically treated obese hypertensive patients (46 males and 83 females, aged 51.95 ± 10.1 years) that came to our observation for a nutritional consultation.

**Methods:**

Study design was retrospective observational. Differences in the prevalence of use of the different antihypertensive drug classes among groups were evaluated with χ^2^ square analysis. Threshold for statistical significance was set at *p* < 0.05.

**Results:**

41.1 % of the study sample was treated with one, 36.4 % with two and the remaining 22.5 % with three or more antihypertensive drugs. In patients under single drug therapy, β-blockers, ACEIs and ARBs accounted each for about 25 % of prescriptions. The prevalence of use of β-blockers was about sixfold higher in females than males. Diuretics were virtually never used in monotherapy regimens but were used in more than 60 % of patients on dual antihypertensive therapy and in all patients assuming three or more drugs. There was no significant difference in the prevalence of use of any of the aforementioned drugs among patients with obesity of type I, II and III or between patients with or without metabolic syndrome.

**Conclusions:**

Our data show that no first choice protocol seems to be adopted in clinical practice for the treatment of obesity-related hypertension. Importantly, physicians do not seem to differentiate drug use according to the severity of obesity or to the presence of metabolic syndrome or to avoid drugs known to detrimentally affect body weight and metabolic profile in general population.

## Background

Overweight and obesity are major risk factors for arterial hypertension [1]. Large epidemiological studies showed that the prevalence of hypertension increases with body weight and is almost doubled in frank obesity [1]. Specifically, about 34 % of normal weight patients are hypertensive whereas this percentage rises up to 60 % in overweight and exceeds 70 % in obese patients [2]. Moreover, the majority of the hypertensive patients seen by general practitioners are overweight or obese [2]. Perspective studies also showed that in non-hypertensive subjects, overweight does increase the chance of later developing new arterial hypertension [3].

Mounting evidence supports the idea that different pathogenetic mechanisms are responsible for *obesity*-*related hypertension* and for hypertension of lean subjects [1, 4]. Specifically, the main determinant of hypertension in lean people is peripheral vasoconstriction, whereas *obesity*-*related hypertension* depends on sympathetic nervous system hyperactivation and on the consequent increase in cardiac output and renin and aldosterone release [1, 4]. The mechanism responsible for sympathetic hyperactivation in obesity seems to be related to the release from adipose tissue of substances such as adipokines, inflammatory cytokines and free fatty acids that may activate autonomic neurotransmission either directly or indirectly, by affecting insulin sensitivity [1, 4–6]. Moreover angiotensin-II (Ang II) and aldosterone that raise blood pressure and promote Na^+^ retention, are both synthesized in adipose tissue [1, 7]. Nonalcholic fatty liver disease (NAFDL) [8, 9] that often coexists with obesity, also has a significant role both in activating the renin-angiotensin-aldosterone (RAA) system and in causing insulin resistance. NAFDL may actually represent and independent cardiovascular risk factor [10] that according to current guidelines, can be corrected lifestyle and dietetic treatment [11].

There is still a considerable uncertainty on which should be the best pharmacological approach to treat *obesity*-*related* hypertension and major guidelines do not expressly address this point [12–14]. Because of the above mentioned pathophysiological differences between *lean* and *obesity*-*related* hypertension, it was suggested that drugs targeting the pathogenetic mechanism of *obesity*-*related hypertension* should be preferred in this condition [1, 15]. Specifically, drugs targeting the RAA system could be a rational choice because of Ang II and aldosterone release from the adipose tissue [16]. β-blockers could also be an option because they counteract the sympathetic overactivation occurring in this condition [1]. However, it has been strongly suggested that when prescribing drug therapy in *obesity*-*related* hypertension, the effect of treatment on body weight and metabolic profile should be carefully considered. Indeed, a note of caution has been raised on the use β-blocker and thiazide diuretics because of the possible detrimental effect of these drugs on body weight and metabolic control [17–19]. The scenario is even more complicated when multiple antihypertensive drugs are required as very often happens in patients with *obesity*-*related hypertension* because of the poor responsiveness of this disease to single drug therapy [1, 20]. The detrimental effect on metabolism and body weight of selected antihypertensive drugs is, indeed, greatly increased when they are used in combinations as, for instance, in the case of thiazide diuretics and β-blockers [21]. In the absence of guideline directions it is unclear how, in clinical practice, these safety concerns influence the choice of antihypertensive therapy for obese patients and whether, because of these concerns, different drugs are used in people with different degrees of obesity. Therefore, in the present paper, we performed a retrospective study on a cohort of pharmacologically-treated obese patients that came to our observation for a nutritional consultation, with the aim of identifying which antihypertensive drugs were more often used in *obesity*-*related hypertension* in a real clinical context.

## Methods

### Study design

This was a retrospective study. Study sample was composed of 129 obese hypertensive patients (BMI ≥ 30) that came to our observation at the Physiology Nutrition Unit of the Federico II University of Naples for a dietitian advice. Only patients with uncomplicated arterial hypertension were included in the study whereas those with angina, arrhythmias or heart failure were excluded. Because of the retrospective design of the study ethical approval was waived according to current Italian legislation (Agenzia Italiana del Farmaco, Determinazione 20 Marzo 2008, Gazzetta Ufficiale della Repubblica Italiana n° 76, 31-3-2008). From the medical records of these patients we retrieved information on age, sex, systolic and diastolic blood pressure and the complete medical history including the list of all the drugs taken at the time of evaluation. In addition, we recollected anthropometric and body composition data that are routinely recorded during patient evaluation at our unit including height, body weight (BW), waist circumference (WC), total (TBW%) and extracellular (ECW%) water and fat (FM%), fat free (FFM%) and muscle mass (MM% of FFM). Body composition was assessed by bioelectrical impedance analysis using a tetrapolar, 50 kHz bioelectrical impedance analyzer (BIA 101 RJL, Akern Bioresearch, Firenze, Italy) [22]. Visceral adiposity was estimated by measuring the visceral adiposity index (VAI), a validated indicator of visceral fat mass [23], using the following equations:

$$ FemaleVAI=\left(\frac{WC}{36.58+\left(1.89\times BMI\right)}\right)\times \left(\frac{TG}{0.81}\right)\times \left(\frac{1.52}{HDL}\right) $$

$$ MaleVAI=\left(\frac{WC}{39.68+\left(1.88\times BMI\right)}\right)\times \left(\frac{TG}{1.03}\right)\times \left(\frac{1.31}{HDL}\right) $$

Patients were classified in two groups according to whether their VAI values were above or below the cut-off values that Amato et al. [24] identified as cardiovascular risk discriminants in Caucasian. Because these values differ in different age groups, we first stratified patients according to their age, then we attributed each of the patients of each age group either to the high or low cardiovascular risk VAI group and, then we pooled altogether people of different age either in the group with VAI below or in the group with VAI above the cutoff value.

Blood chemicals measurements including glycemia, total HDL- and LDL-cholesterol, serum albumin and total protein and transaminases were also obtained.

### Statistical analysis

Statistical analysis was performed with the IBM Statistical Package for Social Science (SPSS) Advanced Statistics software (release 20.0) (Armonk, New York, USA). Continuous data were examined for normality with the Shapiro-Wilk test. Normally distributed data are reported as mean ± standard deviation whereas the median with the 25–75 percentiles is shown for not normally distributed and categorical data.

Patients were classified based on the number of anthypertensive drugs that they were treated with (one, two and three or more) and on the pharmacological class these drugs belonged to (ACEIs, ARBs, Ca^2+^-channel antagonists, β-blockers and diuretics). Student’s t test and Mann-Whitney U test were used for two group comparisons of normally and not-normally distributed data, respectively. Differences in the prevalence of the different antihypertensive drug classes in males and females were evaluated with χ^2^ square analysis. Threshold for statistical significance was set at *p* < 0.05.

## Results

### Characteristics of the study sample

Study sample consisted of 129 patients (aged 51.95 ± 10.1 years) with obesity-related hypertension all pharmacologically treated. 46 patients were males and the remaining 83 were females (36 in premenopausal and 47 in postmenopausal status). Table 1 reports the mean anthropometric, body composition and biochemical data of the study population. 59 patients had class I, 34 class II and 36 class III obesity. 41.1 % of the study sample received a single antihypertensive drug, 36.4 % a combination of two antihypertensive drugs and the remaining 22.5 % three or more antihypertensive drugs. No significant difference was observed in the age of the patients one, two and three or more drugs (Table 2). 16 patients were also treated with lipid-lowering agents, 4 with antidiabetic drugs and 5 with both antidiabetic and lipid-lowering drugs.

**Table 1:** Anthropometric and metabolic data of the whole patient cohort and of patients taking only antihypertensive drugs

	Whole patient cohort			Patients not taking antidiabetic and/or lipid lowering drugs		
	All patients (*n* = 129)	Males (*n* = 46)	Females (*n* = 83)	All patients (*n* = 104)	Males (*n* = 30)	Females (*n* = 74)
Age (yr)	51.95 ± 10.1	51.4 ± 9.6	52.3 ± 10.5	50.9 ± 10.1	49.4 ± 10.2	51.5 ± 10.1
Weight (kg)	95.8 ± 13.8	102.7 ± 12.3	91.9 ± 13.1**	94.4 ± 13.2	100.2 ± 11.9	92.0 ± 13.1*
Height (cm)	161.8 ± 9.4	171.6 ± 6.7	156.4 ± 5.5*	160.6 ± .8.5	1.7 ± .05	160 ± 5.7**
Systolic Arterial Pressure (mmHg)	130.0 (125–140)	130.0 (125–140)	130.0 (125–140)	130.0 (125–140)	132.0 (128.8–140.0)	130.0 (125–140)
Diaystolic Arterial Pressure (mmHg)	85.0 (75.0–90.0)	85.0 (80.0–91.3)	85.0 (75.0–90.0)	85.0 (75.0–90.0)	85.0 (80.0–91.3)	85.0 (75.0–90.0)
WC (cm)	112.8 ± 10.5	116.1 ± 9.3	110.9 ± 10.7*	111.8 ± 9.8	114.3 ± 8.3	110.7 ± 10.3
BMI (kg/m^2^)	35.5 (32.4–40.7)	33.7 (32.0–37.6)	36.7 (33.5–42.0)*	35.7 (32.4–40.6)	33.1 (31.8–36.8)	36.9 (33.5–41.5)*
VAI	1.9 (1.2–2.8)	2.1 (1.4–3.1)	1.8 (1.2–2.6)	1.8 (1.2–2.6)	2.0 (1.4–3.5)	1.8 (1.2–2.5)
TBW%	44.9 ± 5.6	50.6 ± 2.9	41.7 ± 3.9**	44.3 ± 5.4	50.8 ± 2.9	41.7 ± 3.8**
ECW%	43.0 ± 4.4	41.4 ± 4.1	43.9 ± 4.4**	43.1 ± 4.5	40.8 ± 3.7	44.1 ± 4.5**
FM%	39.8 ± 7.5	32.5 ± 4.3	43.8 ± 5.6**	40.3 ± 7.6	31.6 ± 4.6	43.8 ± 5.4**
MM%	42.3 ± 8.4	45.0 ± 8.6	40.8 ± 7.9	42.2 ± 8.2	46.2 ± 8.2	40.6 ± 7.6
Plasma Glusose (mg/dl)	98.0 (91.0–107.0)	101.0 (93.5–112.0)	96.0 (88.0–107.0)	96.5 (88.8–106.0)	99.0 (92.0–107.0)	95.0 (87.0–105.5)
Total Cholesterol (mg/dl)	201.0 ± 40.3	196.8 ± 44.1	203.3 ± 38.1	204.8 ± 38.9	210.3 ± 41.7	202.6 ± 37.9
HDL Cholesterol (mg/dl)	49.0 ± 11.4	41.7 ± 9.4	53.0 ± 10.5**	49.8 ± 11.5	41.5 ± 9.4	53.1 ± 10.6**
LDL Cholesterol (mg/dl)	134.2 ± 38.1	118.1 ± 34.8	143.6 ± 37.4	127.9 ± 36.1	133.1 ± 39.7	125.8 ± 34.6
Triglycerides (mg/dl)	131.0 (92.0–169.5)	143.0 (103.3–211.8)	118.0 (85.0–158.0)*	123.0 (88.3–168.8)	148.5 (102.3–227.5)	116.5 (82.5–154.3)*
Triglycerides/HDL ratio	2.7 (1.7–3.8)	3.5 (2.3–5.3)	2.3 (1.5–3.1)**	2.5 (1.6–3.5)	3.5 (2.3–5.8)	116.5 (82.5–154.3)**
SGOT	23.0 (18.0–29.5)	26.0 (21.8–36.0)	21.0 (17.0–25.0)**	23 (18–19)	28.0 (22.0–36.0)	21.0 (17.0–25.0)**
SGPT	28.0 (19.0–43.5)	43.0 (28.0–61.3)	22.0 (16.0–31.0)**	27 (18.5–41)	43.0 (28.0–61.0)	22.0 (16.8–31.0)**

*p < 0.05 vs males

**p < 0.01 vs males

**Table 2:** Prevalence of use of different antihypertensive drug classes in patients treated with one, two or three or more antihypertensive drugs

Whole patient cohort				Patients not taking antidiabetic and/or lipid lowering drugs		
*Patients treated with a single antihypertensive drug*						
	All patients	Male	Female	All patients	Male	Female
	(*n* = 53; 50.0 ± 10.4 yr)	(*n* = 16; 49.6 ± 10.9 yr)	(*n* = 37; 50.2 ± 10.3 yr)	(*n* = 45; 50.0 ± 10.4 yr)	(*n* = 11; 48.3 ± 12.2 yr)	(*n* = 34; 50.2 ± 11.3 yr)
β-blockers	15 (28.3)	1 (6.3)	14 (37.8)*	12 (26.7)	------	12 (35.3)*
ACEIs	15 (28.3)	8 (50.0)	7 (18.9)*	13 (28.9)	6 (54.5)	7 (20.6)*
ARBs	13 (24.5)	5 (31.3)	8 (21.6)	12 (26.7)	4 (36.4)	8 (23.5)
CCBs	7 (13.2)	2 (12.5)	5 (13.5)	6 (13.3)	1 (9.1)	5 (14.7)
Diuretics	1 (1.9)	------	1 (2.7)	1 (2.2)	------	1 (2.9)
α1 adrenergic blockers	2 (3.8)	------	2 (5.4)	1 (2.2)	------	1 (2.9)
*Patients treated with two antihypertensive drugs*						
	All patients	Male	Female	All patients	Male	Female
	(*n* = 47; 53.1 ± 9.5 yr)	(*n* = 15; 51.7 ± 8.3 yr)	(*n* = 32; 53.8 ± 10.1 yr)	(*n* = 38; 52.0 ± 8.7 yr)	(*n* = 10; 49.9 ± 9.1yr)	(*n* = 28; 52.8 ± 8.7 yr)
β-blockers	12 (25.5)	3 (20.0)	10 (31.3)	10 (26.3)	1 (10.0)	9 (32.1)
ACEIs	19 (40.4)	9 (60.0)	10 (31.3)	14 (36.8)	7 (70.0)	7 (25.0)*
ARBs	23 (48.9)	5 (33.3)	18 (56.3)	19 (50.0)	3 (30.0)	16 (57.1)
CCBs	8 (17.0)	1 (6.7)	7 (21.9)	6 (15.8)	1 (10.0)	5 (17.9)
Diuretics	30 (63.8)	11 (73.3)	19 (59.4)	26 (68.4)	8 (80.0)	18 (64.3)
α1 adrenergic blockers	1 (2.1)	------	1 (3.1)	-------	-------	-------
*Patients treated with three or more antihypertensive drugs*						
	All patients	Male	Female	All patients	Male	Female
	(*n* = 29; 53.6 ± 10.4 yr)	(*n* = 15; 52.9 ± 9.6 yr)	(*n* = 14; 54.4 ± 11.5 yr)	(*n* = 21; 51.3 ± 9.6 yr)	(*n* = 9; 50.1 ± 9.8 yr)	(*n* = 12; 52.3 ± 9.8 yr)
β-blockers	18 (62.1)	9 (60.0)	9 (64.3)	12 (57.1)	4 (44.4)	8 (66.7)
ACEIs	6 (20.7)	2 (13.3)	4 (28.6)	6 (28.6)	2 (22.2)	4 (33.3)
ARBs	19 (65.5)	10 (66.7)	9 (64.3)	14 (66.7)	6 (66.7)	8 (61.5)
CCBs	10 (34.5)	9 (60.0)	1 (7.1)**	8 (38.1)	7 (77.8)	1 (8.3)
Diuretics	28 (96.6)	14 (93.3)	14 (100)	21 (100)	9 (100)	12 (100)
α1 adrenergic blockers	4 (13.8)	2 (13.3)	2 (14.3)	3 (14.3)	1 (11.1)	2 (16.7)

The numbers in parentheses represent the percentage of the total of the respective group

*p < 0.05 vs males

**p < 0.01 at χ^2^ test

### Antihypertensive drugs used in monotherapy, dual therapy and in multiple drug therapy

Table 2 reports the prevalence of use of different antihypertensive drug classes in patients treated with one, two and three or more antihypertensive drugs. In patients on monotherapy, no single class was prevalent over the others and β-blockers, ACEIs and ARBs accounted each for about 25 % of prescriptions. Ca^2+^ channel blockers (CCBs) were used in about 13 % and diuretics in about 2 % of patients. A similar pattern of drug use was observed also when patients treated with lipid-lowering or hypoglycemic drugs were excluded from the analysis. When the two sexes were separately examined, a strong gender-related difference emerged only in the prevalence of use of β-blockers that was about sixfold higher in females than males.

The most remarkable difference that we noticed when we compared patients on monotherapy with those treated with two or more drugs was a markedly higher use of diuretics. These drugs were virtually absent in monotherapy regimens but were used in more than 60 % of patients on dual antihypertensive therapy and in all patients assuming three or more drugs. Prevalence of use of CCBs was not significantly different in patients on mono- or dual-therapy whereas it significantly increased in patients treated with three or more drugs. In this group, about 35 % of patients assumed CCBs with a strong difference between sexes. Indeed, more than 60 % of males and less than 10% of females were treated with these drugs. ACEIs and ARBs were used by about 40 % and 50 % of patients under dual therapy, respectively, with a significant sex difference in ACEI prescription that was more prevalent in males than in females (Table 2). Remarkably, in patients treated with three or more drugs, ARBs were used more often than ACEIs.

### Antihypertensive drugs utilization in patients with different degrees of obesity

To establish whether different drugs are prescribed in patients with different degrees of obesity we stratified our patients according to their BMI and compared the prevalence of use of β-blockers, ACEIs, ARBs, CCBs, diuretics and α1 adrenoceptor blockers. As shown in Table 3 we did not find any significant difference in the prevalence of use of any of the aforementioned drugs among patients with type I, II and III obesity.

**Table 3:** Prevalence of use of different antihypertensive drug classes in patients with obesity of class I, II and III

	β-blockers	ACEIs	ARBs	CCBs	Diuretics	α1 adrenergic blockers
Whole population (*n* = 129)	45 (34.9)	39 (30.2)	55 (42.9)	25 (19.4)	60 (46.9)	7 (5.4)
Class I (*n* = 59)	13 (22.0)	25 (42.3)	23 (38.9)	16 (27.1)	26 (44.1)	1 (1.7)
Class II (*n* = 34)	19 (55.9)	6 (17.6)	16 (47.1)	5 (14.7)	18 (52.9)	1 (2.9)
Class III (*n* = 36)	13 (36.1)	8 (22.0)	16 (44.4)	4 (11.1)	16 (47.1)	5 (13.9)
Only antihypertensive (*n* = 104)	35 (33.7)	33 (31.7)	45 (43.3)	20 (18.3)	48 (46.2)	4 (3.7)
Class I (*n* = 46)	10 (21.7)	18 (39.1)	20 (43.5)	12 (26.1)	21 (45.7)	1 (2.2)
Class II (*n* = 29)	14 (48.3)	6 (20.7)	13 (44.8)	4 (13.8)	15 (51.7)	1 (3.4)
Class III (*n* = 29)	11 (37.9)	9 (31.0)	12 (41.4)	4 (13.8)	12 (41.4)	2 (6.8)

The numbers in parentheses represent the percentage of the total of the respective group

Because BMI does not reflect only the amount of fat in the body but it is also influenced by lean tissue mass, it could not represent the best parameter to quantify the the impact of obesity on arterial blood pressure. Recent evidence suggests that specific age-related cut-off values of the visceral adiposity index, a parameter that faithfully represent metabolically active visceral fat [23], could identify people with high cardiovascular risk [24]. Therefore, we compared the use of the different classes of antihypertensive drugs in patients above and below this cut-off value. The results reported in Table 4, did not show any significant difference with the only exception of ARB use that was higher in patients with values of VAI above cut-off. However, this difference was significant only if the whole patient population was considered and not if patients receiving only antihypertensive drugs were evaluated (Table 4).

**Table 4:** Prevalence of use of different antihypertensive drug classes in patients below and above the high cardiovascular risk VAI cutoff value

	β-blockers	ACEIs	ARBs	CCBs	Diuretics	α1 adrenergic blockers
Whole population (*n* = 129)	45 (34.9)	39 (30.2)	55 (42.9)	25 (19.4)	60 (46.9)	7 (5.4)
VAI class 0 (*n* = 70)	25 (35.7)	25 (35.7)	24 (34.3)*	10 (14.3)	33 (47.1)	4 (5.7)
VAI class I (*n* = 59)	20 (33.9)	14 (23.7)	31 (52.5)	15 (25.4)	27 (45.8)	3 (5.1)
Only antihypertensive (*n* = 104)	35 (33.7)	33 (31.7)	45 (43.3)	20 (18.3)	48 (46.2)	4 (3.7)
VAI class 0 (*n* = 60)	18 (30.0)	21 (35.0)	22 (36.7)	9 (15)	29 (48.3)	2 (3.3)
VAI class I (*n* = 44)	17 (38.6)	12 (27.3)	23 (52.3)	11 (25)	19 (43.2)	2 (4.5)

Patients in VAI class 0 have values of VAI below the high cardiovascular risk cutoff value whereas those with values above this cutoff are in VAI class I. The numbers in parentheses represent the percentage of the total of the respective group

*p < 0.05 vs VAI class I

### Antihypertensive drugs used in patients with or without metabolic syndrome

67 (51.9 %) of the 129 patients of the whole population and 49 (47.1 %) of the 104 treated only with antihypertensive drugs had metabolic syndrome according to the ATP III criteria [25]. There was no significant difference in the prevalence of use of β-blockers, ACEIs, ARBs, CCBs, diuretics and α1 adrenoceptor blockers in patients with or without this syndrome neither when we considered the whole patient population or the patients treated only with antihypertensive drugs and no antidiabetic or lipid-lowering drugs (Table 5).

**Table 5:** Prevalence of use of different antihypertensive drug classes in patients with or without metabolic syndrome

	β-blockers	ACEIs	ARBs	CCBs	Diuretics	α1 adrenergic blockers
No metabolic syndrome						
Whole population (*n* = 62)	23 (37.1)	18 (29.0)	27 (43.5)	11 (54.8)	32 (14.5)	2 (3.2)
Only antihypertensive (*n* = 55)	21 (38.2)	15 (27.3)	26 (47.3)	9 (16.4)	29 (52.7)	1 (1.8)
Antihypertensive plus antidiabetic or lidip lowering drugs (*n* = 7)	2 (28)	3 (42.9)	1 (14.3)	2 (28.6)	3 (42.9)	1 (1.8)
Metabolic syndrome						
Whole population (*n* = 67)	22 (32.8)	21 (31.3)	28 (41.8)	14 (40.3)	28 (22.4)	5 (7.5)
Only antihypertensive (*n* = 49)	14 (28.6)	17 (34.7)	19 (38.8)	11 (22.4)	20 (40.8)	3 (6.1)
Antihypertensive plus antidiabetic or lidip lowering drugs (*n* = 18)	8 (44.4)	4 (22.2)	9 (50.0)	3 (16.7)	8 (44.4)	2 (11.1)

The numbers in parentheses represent the percentage of the total of the respective group

## Discussion

In the present study we retrospectively evaluated the utilization of antihypertensive drugs in a cohort of obese hypertensive patients to establish whether in obesity-related hypertension, practitioners preferentially use drugs known not to negatively affect metabolism or body weight. The main finding of our study was that, instead, our obese patients were treated with drugs belonging to all the main antihypertensive drug classes, also including those expected to increase body weight or worsen metabolic profile.

The analysis of patients on monotherapy showed that no single antihypertensive drug was used as first choice with the patients almost equally distributed among those taking β-blockers, ACEIs and ARBs whereas use prevalence of CCBs was only slightly lower. The finding that about 25 % of patients on monotherapy were treated with β-blockers was unexpected. Indeed, although these drugs may counteract the sympathetic hyperactivity that is responsible for *obesity*-*related hypertension*, concerns have been raised on their tolerability in this clinical condition because current evidence suggests that they could increase body weight and worsen metabolic status [1, 17]. Interestingly, almost all of the patients taking β-blockers as single drug therapy were women. A possible explanation of this finding is that β-blockers may cause erectile dysfunction and, therefore, are not well accepted by male patients [26]. Thiazide diuretics were virtually never used as single drugs in our population although they are considered first choices drugs in current guidelines [12, 13]. This suggests that practitioners had the perception that thiazide diuretics should better not be used in obesity and that they modified their prescriptions accordingly. Specifically, published evidence that these diuretics may worsen metabolic profile and cause impotence was probably responsible for keeping low their use in our group of obese patients [18, 26, 27]. While almost never used in single drug therapy, diuretics were perceived as important drugs in multiple-drug therapy as all the patients of our cohort that were treated with three or more drugs took diuretics in various combinations with drugs acting on RAA system and CCBs. An interesting finding was that patients treated with three or more drugs took less ACEIs and more ARBs in comparison with those on monotherapy. This was not unexpected considering that ARBs are often used when patients stop responding to ACEIs because of Ang II escape. Therefore we can hypothesize that patients treated with multiple drugs switched from ACEIs to ARBs sometime before coming to our observation because of acquired drug resistance.

We did not find any significant difference in the prevalence of use of any drug class when comparing patients with obesity class I, II or III. Moreover, there was no significant difference between patients with or without metabolic syndrome. No difference was observed also when comparing patients with high or low values of VAI, with the only exception of a higher prevalence of ARB use in patients with high VAI. However, this difference was significant only when the whole patient population also including those taking lipid lowering or antidiabetic drugs was considered. Therefore, it probably does not reflect different drug choice related to hypertension *per se* but could be dependent on the presence of a concomitant disease such as diabetes. Indeed, ARBs (and ACEIs) could represent first choice drugs in diabetes especially in the presence of renal damage [12]. Collectively, our results seem to suggest that although concerns have been raised on the use of some antihypertensive drugs because of their effect on body weight and metabolism [28–31], the degree of obesity or the presence of its metabolic complications did not influence drug choice in our patients. These data could also suggest that, in our patients, the class of antihypertensive drugs used was not a major determinant either of BMI or of metabolic control. However, our study was not specifically designed to address this question, and further randomized prospective studies will be needed to address this point also considering that available information in the literature is very limited. Specifically, a few studies showed that drugs acting on RAA system and, in particular, ARBs could improve metabolic status in patients with metabolic syndrome [32], decreasing visceral fat accumulation [33] and improving insulin sensitivity and lipid profile [34], whereas visceral adiposity increases the risk of developing adverse metabolic effects upon treatment with β-blockers or thiazide diuretics [35]. Moreover, an important limitation of these studies was the short duration of drug exposure ranging around several weeks. The issue of establishing whether adiposity or metabolic status are affected by specific antihypertensive drug classes in obesity is potentially clinically relevant because, by interfering with these parameters, drug therapy could positively or negatively influence long term prognosis in this condition. Under this respect, it is worth mentioning the recent evidence reported by the Blood Pressure Lowering Treatment Trialists’ Collaboration [36] showing that in obesity-related hypertension the outcome measured as a composite of major cardiovascular events including stroke, coronary heart disease, heart failure, and cardiovascular death, is independent from the class of antihypertensive drugs taken by the patients.

Although our retrospective study was performed on medical records from a single institution, we believe that it is actually representative of a larger population of obese patients. Indeed, our unit is not a primary center for the treatment of hypertension but a nutritional consultation with its catchment area extending across the larger Naples metropolitan region. Therefore, the data on antihypertensive drug treatment that we analyzed in the present study do not reflect the therapeutic choices of physicians working in a single center but those of primary care physicians or of cardiologists taking care of the patients in many other institutions that sent us their patients only for a nutritional advice. A limitation of the study is that, we cannot exclude a selection bias because the patients that we examined were actually sent to our observation by other physicians to further improve their medical treatment. This implies that our study sample could have been theoretically composed by patients taking benefits of higher standard than average medical care.

## Conclusions

In conclusion, we showed that the pharmacological approach to the treatment of *obesity*-*related hypertension* is highly heterogeneous as different drug classes are used either alone or in combination and no first choice protocol seems to be adopted in clinical practice. Importantly, we found no evidence that physicians differentiate drug use according to the severity of obesity, to visceral fat accumulation or to the presence of metabolic syndrome. There is an urgent need of further data to provide informed directions that could help practitioners in choosing the right therapy for hypertensive obese patients. Specifically, well designed randomized trials are needed to establish whether the detrimental effect of some antihypertensive drugs that were observed in general population also occur in obese patients.

## Abbreviations

ACEI: ACE inhibitor
ARB: Angiotensin II receptor blocker
Ang II: Angiotensin-II
BMI: Body mass index
BW: Body weight
CCB: Calcium channel blocker
ECW%: Percent extracellular water
FFM%: Percent fat free mass
FM%: Percent fat mass
MM%: Muscle mass as percent of free fat mass
NAFLD: Nonalcholic fatty liver disease
RAA: Renin-angiotensin-aldosterone
TBW%: Percent total body water
VAI: Visceral adiposity index
WC: Waist circumference
